# Not COVID-19, Don’t Overlook Pneumocystis in Patients on Gefitinib!

**DOI:** 10.3390/curroncol28010094

**Published:** 2021-02-21

**Authors:** Jérémy Barben, Valérie Quipourt, Jérémie Vovelle, Alain Putot, Patrick Manckoundia

**Affiliations:** Geriatrics Department, University Hospital of Dijon, 21000 Dijon, France; valerie.quipourt@chu-dijon.fr (V.Q.); jeremie.vovelle@chu-dijon.fr (J.V.); alain.putot@chu-dijon.fr (A.P.); patrick.manckoundia@chu-dijon.fr (P.M.)

**Keywords:** COVID-19, gefitinib, pneumocystis jirovecii, tyrosine-kinase inhibitor

## Abstract

**Simple Summary:**

COVID-19 pneumonia can be confused with Pneumocystis jirovecii pneumonia (PJP) on computed tomography and is a source of misdiagnosis. This can lead to mistreatment and an increased risk of mortality. Gefitinib, a tyrosine kinase inhibitor anti-EGFR used in cancer therapy and corticosteroids, could increase the risk of PJP.

**Abstract:**

An 82-year-old woman treated for advanced lung cancer with gefitinb was admitted to the emergency unit complaining of dyspnea. Chest computed tomography found abnormalities classified as possible diffuse COVID-19 pneumonia. RT-PCR for Sars-Cov-2 was twice negative. PCR for Pneumocystis jirovecii was positive on bronchoalveolar lavage. The final diagnosis was Pneumocystis jirovecii pneumonia. Therefore, physicians must be careful not to misdiagnose COVID-19, especially in cancer patients on small-molecule therapeutics like gefitinib and corticosteroids.

## 1. Introduction

Since December 2019, the medical community has been dealing with the SARS-CoV-2 pandemic. We report here an original case where the focus on an erroneous diagnosis of COVID-19 delayed the management of a Pneumocystis jirovecii infection acquired during treatment with gefitinb, a tyrosine kinase inhibitor (TKI), for lung cancer.

## 2. Case Presentation

An 82-year-old woman complaining of dyspnea was admitted to the emergency unit of a French university hospital in October 2020, during the SARS-CoV-2 pandemic. She had been diagnosed with locally advanced lung adenocarcinoma (T2N2M0 with EGFR L858R activating mutation) in June 2020. The patient refused any surgical or radiotherapy treatment. Osimertinib was prescribed as a first-line treatment, but after one month, the woman developed pneumonitis whose origin, after multidisciplinary consultation, was possibly attributed to osimertinib. Corticosteroid therapy with betamethasone was initiated at 6 mg and then rapidly tapered, resulting in good resolution of the pneumonitis. After six weeks, when betamethasone had been reduced to a dose of 1 mg, corticosteroid therapy was switched to hydrocortisone 20 mg. Osimertinib was replaced by gefitinib after two weeks of corticosteroid therapy. No antifungal prophylaxis was initiated. After the switch to gefitinb and low-dose hydrocortisone, tolerance was found to be excellent, and the control PET (positron emission tomography) scan performed at the end of September 2020 showed a complete metabolic response and the absence of pneumonitis. 

**Figure 1:**
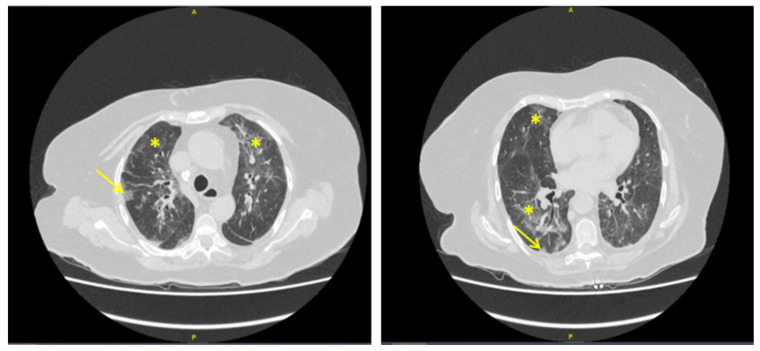
Chest computed tomography (CT) of an 82-year-old woman with advanced lung adenocarcinoma successfully treated with gefitinib and admitted for dyspnea two days previously. Bilateral ground glass opacities (*), and subpleural condensation (→) were interpreted as possible diffuse COVID-19 pneumonia. Note the absence of crazy paving aspect. RT-PCR on nasopharyngeal swab was negative for Sars-Cov-2 twice. A final diagnosis of Pneumocystis jirovecii pneumonia on gefitinib (a tyrosine kinase inhibitor) was retained after positive blood (1-3)-Beta-D-glucane and PCR for P. jirovecii on bronchoalveolar lavage.

The woman’s current complaint started a couple of days before with dyspnea, delirium, and fever. Diffuse rhonchi were found on pulmonary auscultation. The results of the blood analysis were as follows: leukocytes 8.1 × 10^9^ cells/L, neutrophils 7 × 10^9^ cells/L, lymphocytes 0.46 × 10^9^ cells/L, platelets 398 × 10^9^ cells/L, hemoglobin 10.1 g/dL, and C-reactive protein 341 mg/L. The chest computed tomography (CT) scan showed multiple bilateral apical and peri-hilar ground glass opacities (GGO) associated with subpleural condensation. These findings were classified as possible diffuse COVID-19 pneumonia (Figure 1) by the radiologist [1]. However, the RT-PCR (Reverse Transcription Polymerase Chain Reaction) for SARS-CoV-2 performed on nasopharyngeal swab on admission, two days after the symptoms began, was negative. A diagnosis of COVID-19 was nonetheless initially retained and the patient received dexamethasone recommended by the Recovery trial [2,3]. When the initial course was not favorable, oxygen therapy was increased, and a second RT-PCR for Sars-Cov-2 was negative. We performed a blood (1-3)-Beta-D-glucan test, which was positive at 90 pg/mL (negative <60 pg/mL, equivocal between 60 and 80 pg/mL, positive above 80 pg/mL). PCR for Pneumocystis jirovecii was positive on bronchoalveolar lavage. The final diagnosis was Pneumocystis jirovecii pneumonia (PJP), and the patient responded favorably to treatment with sulfamethoxazole-trimethoprim (SMX-TMP). After discussion with the pulmonologists, gefitinib was considered to be potentially responsible for the infection.

## 3. Discussion

In this case, the physicians initially focused on the findings of the chest CT. In COVID-19, the typical features of CT imaging are bilateral, multifocal rounded, and peripheral GGO, with or without consolidations or visible intralobular lines, also known as “crazy paving” [4]. GGO is a common CT sign in interstitial lung disease [5]. There are numerous etiologies, and Pneumocystis is a well-known cause of CT abnormalities in immunocompromised patients [6,7]. PJP has been reported in some non-small-cell lung cancer patients treated with radiochemotherapy or prolonged corticosteroid therapy [8,9]. PJP mainly occurs in patients with defective CD4 T-lymphocyte–macrophage crosstalk [10]. Our patient was obviously immunocompromised with profound lymphopenia, which could be explained by both the cancer and immunosenescence [11]. Here, the role of corticosteroid therapy at the beginning of the infection cannot be excluded, especially since the patient was not taking SMX-TMP prophylaxis. However, the rapid decrease and the very low dose of hydrocortisone taken for several weeks led to the hypothesis of the imputability of the TKI. Several TKIs such as idelalisib are known to generate a higher risk of PJP [7,12]. Most invasive fungal infections described in the literature involve TKIs inhibiting the PI3K pathway, while gefitinib inhibits the Ras signaling pathway by inhibiting EGFR autophosphorylation [13,14]. While gefitinib has not been associated with more frequent infections, particular attention should be paid to such events in patients treated with TKIs [10]. At least, previous prolonged administration of corticosteroids may have contributed to the development of PJP. Nonetheless, this is the first case of PJP diagnosed in a patient treated with gefitinib. 

## 4. Conclusions

Physicians must be careful not to misdiagnose COVID-19, especially in cancer patients on small-molecule therapeutics. Prolonged corticosteroids and TKI anti-EGFR like gefitinib could increase the risk of PJP. SMX-TMP prophylaxis could be routinely discussed for patients on TKIs, especially those with corticosteroid intake.
